# The dynamic distribution of *duck Tembusu virus* in the spleen of infected shelducks

**DOI:** 10.1186/s12917-019-1860-6

**Published:** 2019-04-11

**Authors:** Xuejing Sun, Enxue Liu, Adeela Iqbal, Taozhi Wang, Xindong Wang, Abdul Haseeb, Nisar Ahmed, Ping Yang, Qiusheng Chen

**Affiliations:** Ministry of Education Joint International Research Laboratory of Animal Health and Food Safety, College of Veterinary Medicine, Nanjing Agricultural University, Wei gang No.1, Nanjing, 210095 Jiangsu Province China

**Keywords:** *Duck Tembusu virus*, Spleen, Dynamic distribution

## Abstract

**Background:**

*Duck Tembusu virus* (DTMUV) is a novel member of *Flavivirus*. The isolated and purified DTMUV strain XZ-2012 was used as a strain model, to intramuscularly inject the six-month egg-laying shelducks with the infective dose of 10^4^TCID_50_. The dynamic distribution of the virus in spleen at different time post-infection (pi) was studied using RT-PCR, RT-qPCR, ELISA, immunofluorescence and transmission electron microscopy (TEM).

**Result:**

The results showed that the virus occurred in the spleen after 2 hpi and lasted up to 18 dpi. The registered viral load increased from 2 hpi to 3 dpi, and then it diminished from 6 dpi to 18 dpi with a slight rise at 12 dpi. From 2 hpi to 6 dpi the DTMUV particles were mostly distributed in the periellipsoidal lymphatic sheath (PELS) of spleen white pulp, few being found in the sheathed capillary. From 9 dpi to 18 dpi, the DTMUV particles were migrating into periarterial lymphatic sheaths (PALS) around the central artery through the red pulp. Under TEM, the virus particles could be observed mostly in lymphocytes and macrophages.

**Conclusion:**

It was suggested that DTMUV invaded lymphocytes and macrophages of the spleen at 2 hpi and replicated significantly from 1 dpi to 3 dpi, being eliminated from 9 dpi to 18 dpi. This is the first study on the dynamic distribution of DTMUV from invasion to elimination in duck spleen conducted by molecular and morphological methods. It could provide theoretical basis for the occurrence, development and detoxification of the virus in the organs of the immune system.

## Background

In April 2010, an infectious disease of egg-laying ducks outbroke in the region of southeastern coastal provinces of China, caused by a novel type of *Flavivirus* infection, named *duck Tembusu virus* (DTMUV) [1–5]. DTMUV is a positive sense single-stranded RNA enveloped virus from *Flavivirus* genus, *Flaviviridae* family. The DTMUV genome is approximately 11 kb in length, consisting of a 5′-terminal non-coding region and a 3′-terminal non-coding region and a middle open reading frame. This open reading frame encodes 3 structural proteins: the capsid protein (C), the membrane protein precursors (PrM) and the envelope protein (E), and 7 non-structural proteins (NS1, NS2A, NS2B, NS3, NS4A, NS4B and NS5) [6, 7]. The envelope protein is the main structural protein that forms the virus envelope and contains multiple epitopes, being the main antigen protein that triggers the host’s immune response [8]. The mature virion could be observed by transmission electron microscopy (TEM), having the nucleic acid in the center and an envelope with the diameter of 40 to 60 nm [9]. There is a high homology among various DTMUV strains and other *Flaviviruses* [10].

DTMUV exhibits a wide range of natural host species, including egg-laying ducks [3], meat-type ducks [11, 12], chickens [13, 14], geese [15–17] and mice [18, 19], etc. Moreover, it can proliferate in duck embryo, chicken embryo, duck and chicken embryo fibroblast, baby hamster kidney cell 21 (BHK-21) and Vero cell [20]. At present, the common diagnostic methods used in the laboratory are serological tests such as enzyme-linked immunosorbent assay (ELISA) [21], the neutralization test, and the molecular biological methods such as reverse transcription-polymerase chain reaction (RT-PCR), reverse transcription-loop-mediated isothermal amplification (RT-LAMP) and real-time quantitative polymerase chain reaction (RT-qPCR) [22].

*Flaviviruses* Can lead to viremia through vascular system and accumulate firstly into the spleen [23–25]. The spleen exhibited higher viral loads compared to other organs [22, 26]. In birds, the spleen is the largest secondary lymphatic organ and plays an important role in restraining the circulating pathogens. The duck spleen composes of white pulp and red pulp without marginal zone. The red pulp consists of splenic cord and splenic sinus. The white pulp is composed of circular ellipsoids around the sheathed capillary that has cuboidal-shaped endothelial cells, periellipsoidal lymphatic sheaths (PELS), periarterial lymphatic sheaths (PALS) around the central artery, and splenic nodules. The ellipsoid consisted of several layers of supporting cells. In particular, the duck spleen has a blood-spleen barrier (BSB), as a kind of immune barrier [27]. Whether BSB blocks the spreading of DTMUV, affecting its dynamic distribution, it is still unclear. In this experiment, the isolated and purified strain XZ-2012 was used as strain model, and the changes in virus distribution and content in infected duck spleen from invasion to elimination was investigated. It could provide a theoretical basis for the dynamic distribution of this virus in the immune organ and the relationship between the virus and the immune barrier.

## Results

### DTMUV strain XZ-2012 amplification

**Fig. 1:**
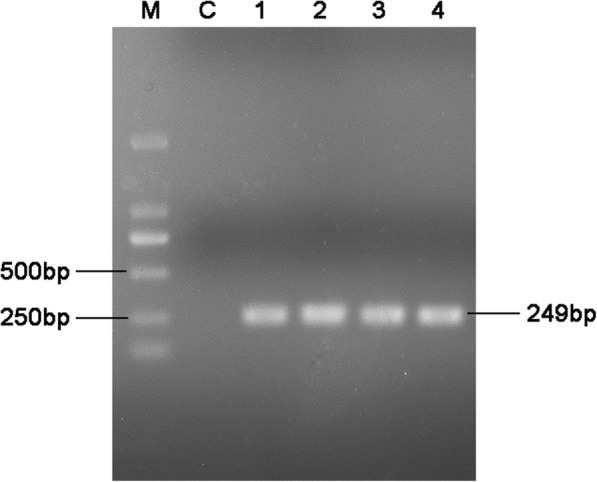
RT-PCR results of duck embryos allantoic fluid. M: DL 2000 bp DNA marker; C: Duck embryo allantoic fluid of control; 1–4: Duck embryo allantoic fluid of treatment

**Fig. 2:**
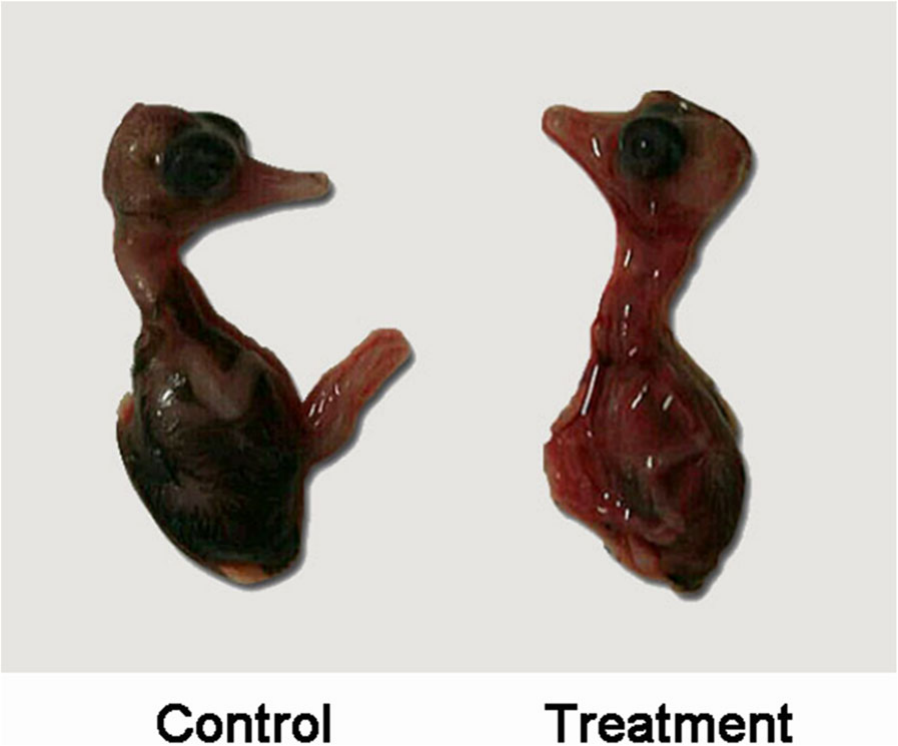
Control duck embryo and infected duck embryo. Infected duck embryo is systemic hyperemic and hemorrhagic

RT-PCR products of duck embryo allantois fluid were detected by agarose gel electrophoresis. The positive band size was approximately 249 bp, which was in accordance with the expected size (Fig. 1). Hyperemia and hemorrhage were present in the whole body of the infected duck embryos (Fig. 2).

### DTMUV TCID_50_

The TCID_50_ results were calculated using the Reed-Muench method. TCID_50_ was 10^4.7^/mL.

### Qualitative detection of DTMUV in blood and spleen

**Fig. 3:**
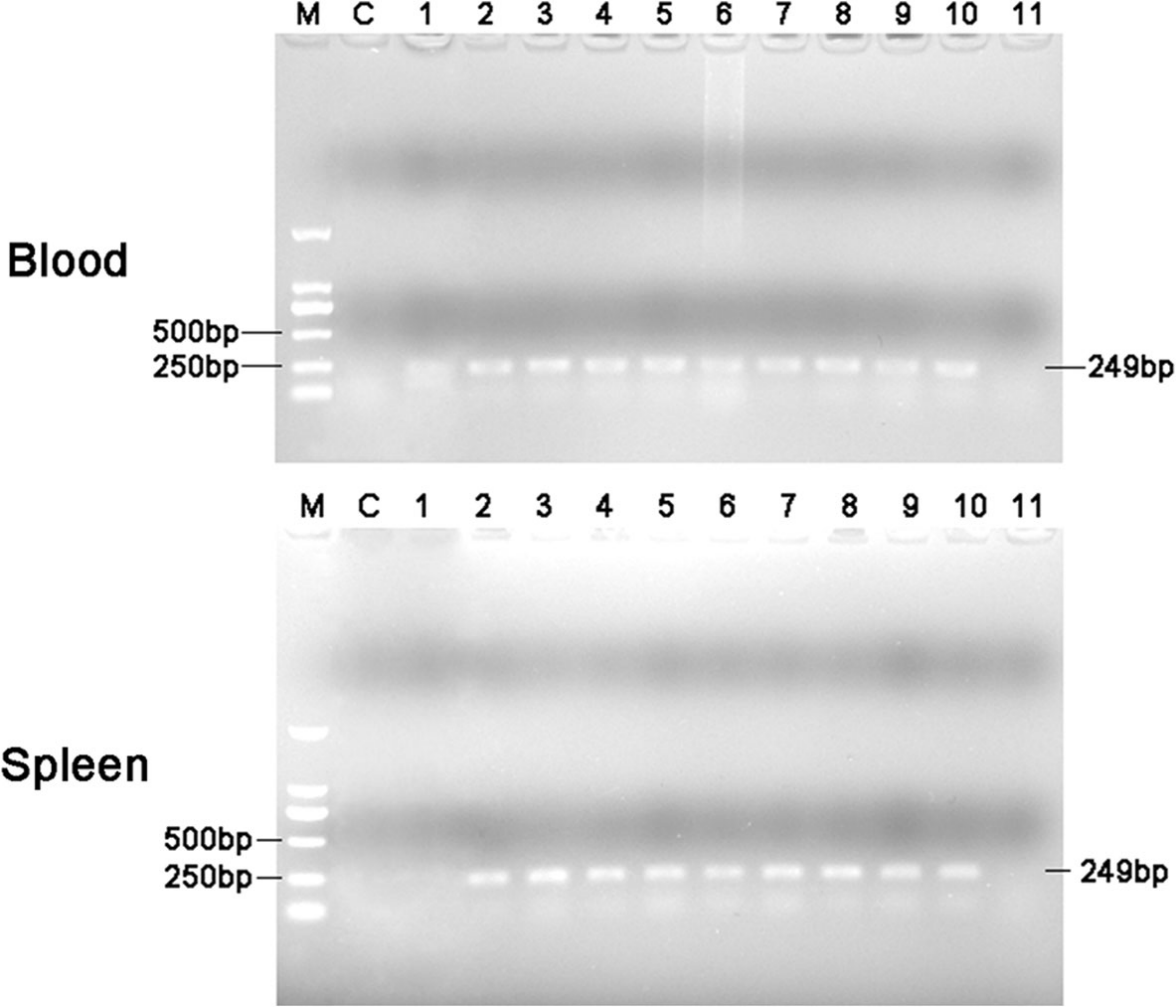
RT-PCR results of duck blood and spleen. M: DL 2000 bp DNA marker; C: control group; 1–11: The time course of infection from 1 h to 24d

RT-PCR products of duck blood and spleen were detected by agarose gel electrophoresis. It was found that the DTMUV was present in blood from 1 hpi to 18 dpi, and in spleen from 2 hpi to 18 dpi. The control group was negative (Fig. 3). The results of the qualitative ELISA were consistent with those of RT-PCR.

### Quantitative detection of DTMUV in the spleen

#### RT-qPCR

**Fig. 4:**
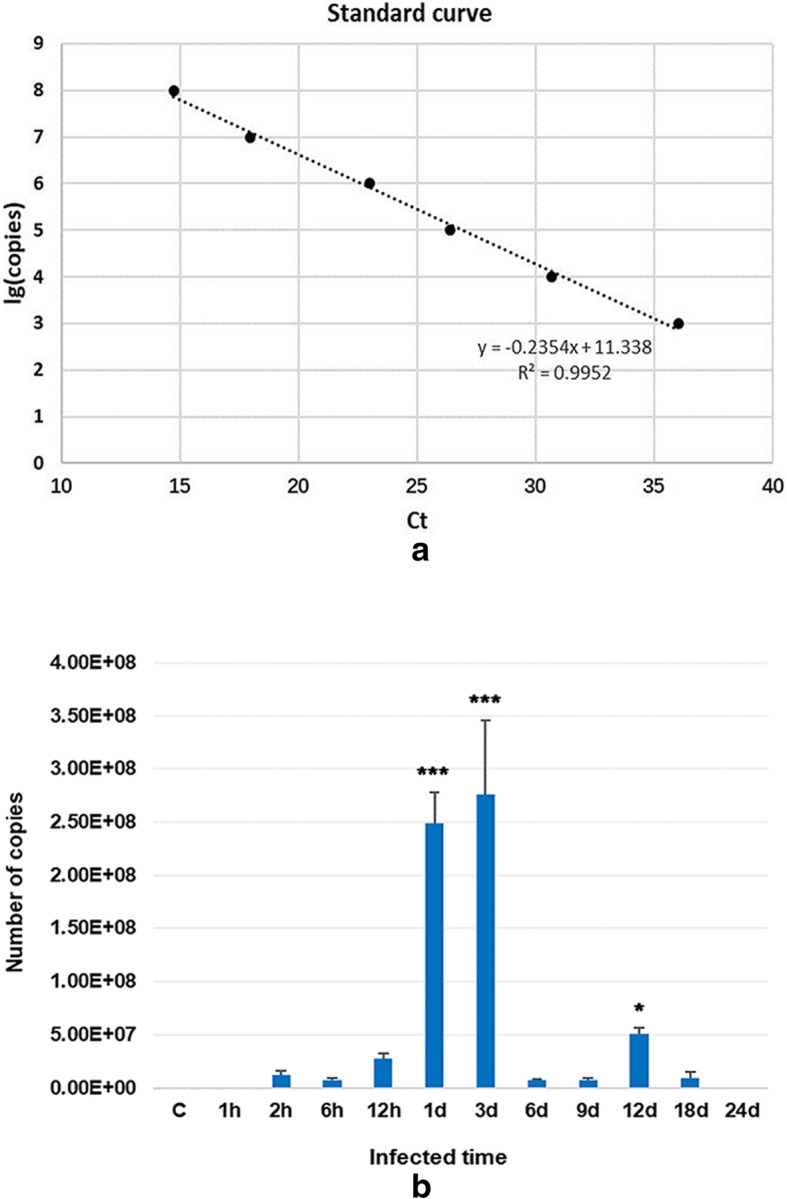
Virus RT-qPCR results. **a** The linear formula of standard curve is y = 11.338–0.2354x. X-axis represents the Ct value of the detected samples, Y-axis represents the usual logarithm of the initial concentration of samples. **b** The histogram of the mean ± SEM of virus gene copy. The copies number of virus genes is increasing from 2hpi, then decreases. There is a highly significant difference (*P*<0.001) at 1dpi and 3dpi and a significant difference (*P*<0.05) at 12dpi compared to that of the control (C)

XZ-2012 virus concentrations in the spleen were calculated according to a standard curve that had a linear relationship with Ct value in the range from 10^3^ copies to 10^8^ copies. The results showed that the Ct value and the number of copies from 2 hpi to 18 dpi were all in the detectable range. The number of viral genes copies increased slowly between 2 hpi and 12 hpi, and significantly at 1 dpi and 3 dpi, then rapidly decreased, while registered a slight rise at 12 dpi. The number of copies at 1 hpi and 24 dpi was too low to be detected (Fig. 4).

#### Elisa

**Fig. 5:**
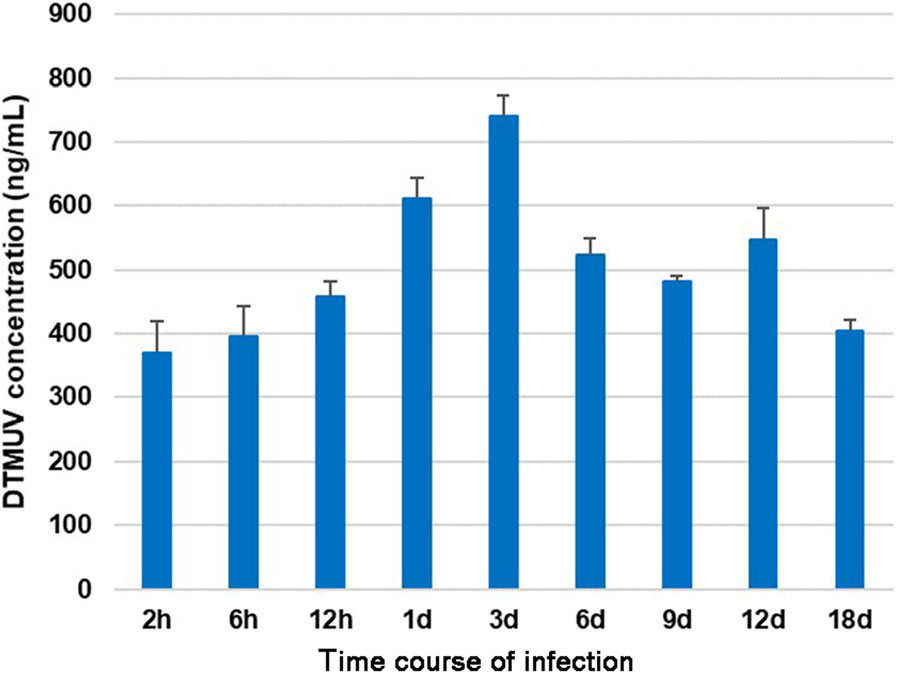
The histogram of the mean ± SEM of virus concentration. The virus concentration is increasing from 2hpi, and has a highest value at 3dpi, then decreases, while has a weaker rise at 12dpi

The concentrations of XZ-2012 strain in the spleen from 2 hpi to 18 dpi were detected by ELISA, which showed a similar change trend to that of the viral gene. The virus concentration was increasing between 2 hpi and 3 dpi and reached the highest level at 3 dpi, then decreased, while had a slight rise at 12 dpi (Fig. 5).

### DTMUV localization in the spleen

#### Immunofluorescence

**Fig. 6:**
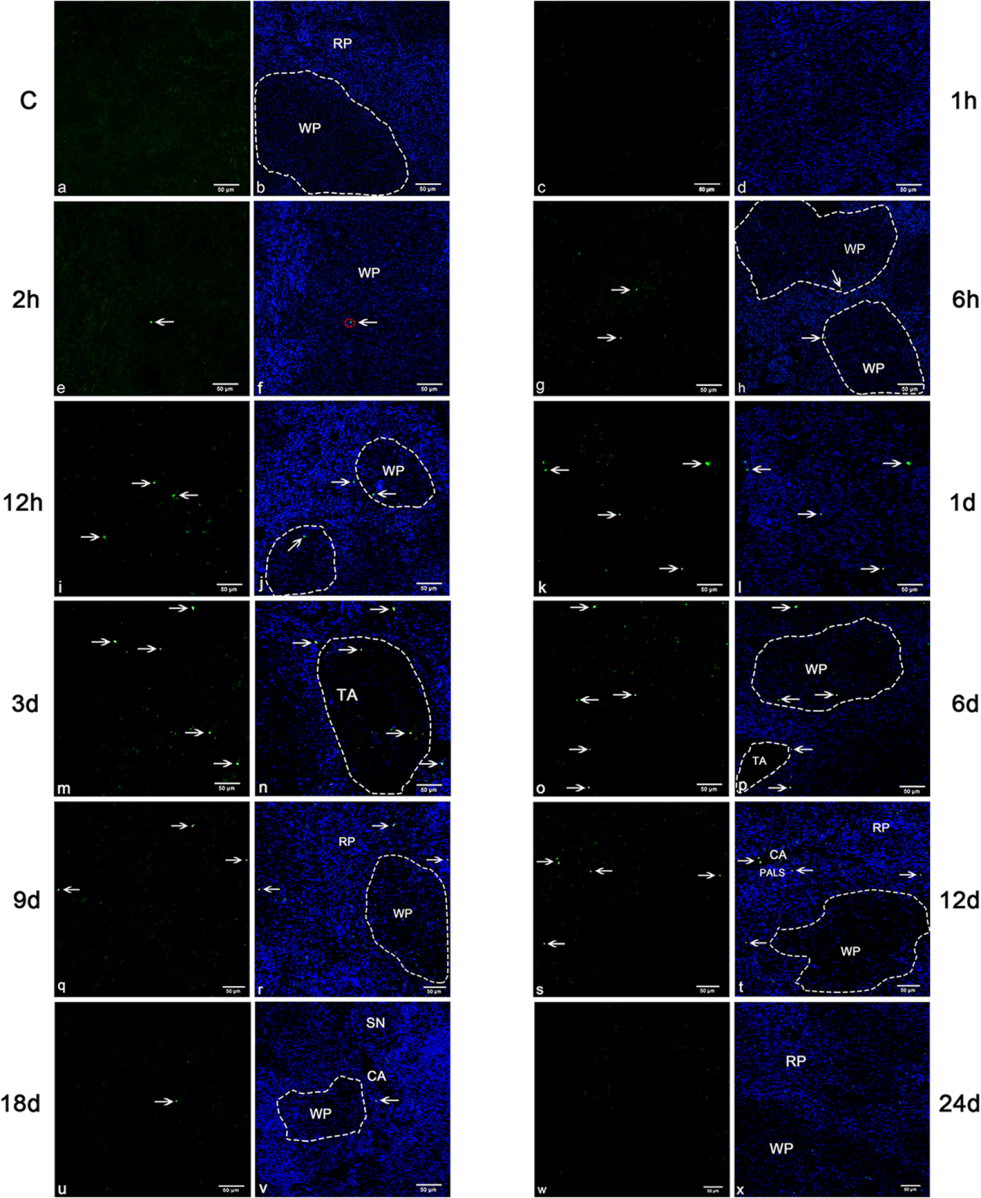
Immunofluorescence results in duck spleens. The virus was detected in spleen between 2 hpi to 18 dpi and dispersedly distributed as dot-like particles (→), not detected at 1 hpi and 24 dpi. At 2hpi, the particles are found in the sheathed capillary (red dotted borders in Fig. 6f) and the periphery of white pulp (white dotted borders). Between 6hpi to 6dpi, the virus particles were mostly distributed in the periphery of white pulp (white dotted borders). Some virus particles are also found on the wall of the trabecular artery vessel and in the periphery of the trabecular artery (white dotted borders in Fig. 6n and p) at 3dpi and 6pi. Between 9dpi to 18dpi, the virus particles are mainly observed in the red pulp and periarteriolar lymphatic sheaths (PALS) around the central artery. Bar = 50 μm. RP: red pulp, WP: white pulp, TA: trabecular artery, CA: central artery, PALS: periarteriolar lymphatic sheaths

**Fig. 7:**
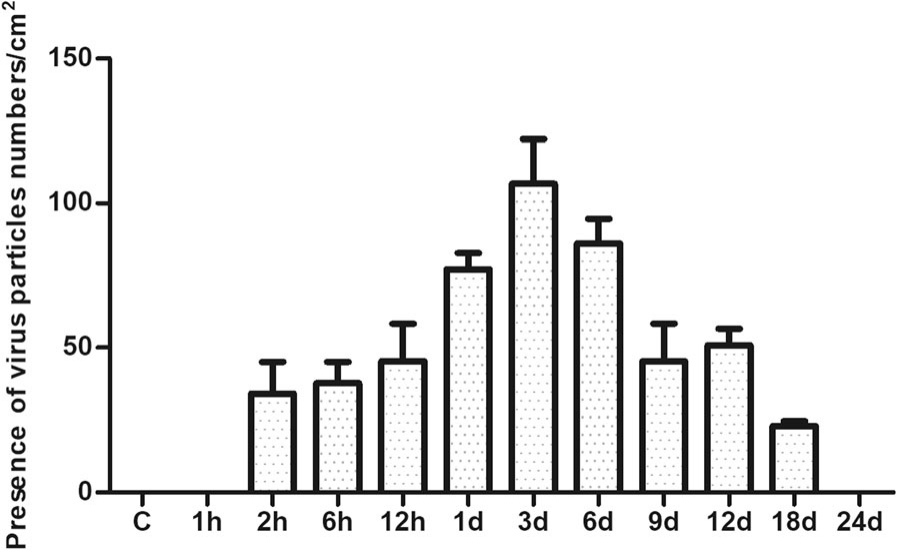
The count of presence of virus particles

The immunofluorescence results revealed the location of XZ-2012 DTMUV in spleen at different times. It was observed that the positive reactions of the virus were detected in spleen from 2 hpi to 18 dpi and appeared as dot-like particles, while not being detectable in the spleen at 1 hpi or 24 dpi. The positive particles were mostly distributed in the region of white pulp from 2 hpi to 6 dpi. The positive particles were found in the PELS and the sheathed capillary surrounded by ellipsoid and at 2 hpi, and mostly distributed in the PELS from 6 hpi to 6 dpi. Furthermore, some positive particles were also found on the wall of the trabecular artery vessel and in the periphery of the trabecular artery at 3dpi and 6pi after infection. At 9 dpi, the positive particles were mainly observed in the red pulp. From 12 dpi to 18 dpi, the positive particles were mostly distributed in periarteriolar lymphatic sheaths (PALS) around the central artery (Fig. 6). Calculated with Image-pro plus software, the number of positive particles was increased first and then decreased after infection, reaching to the peak at 3dpi. Few particles were found at 18 dpi (Fig. 7).

#### Tem

**Fig. 8:**
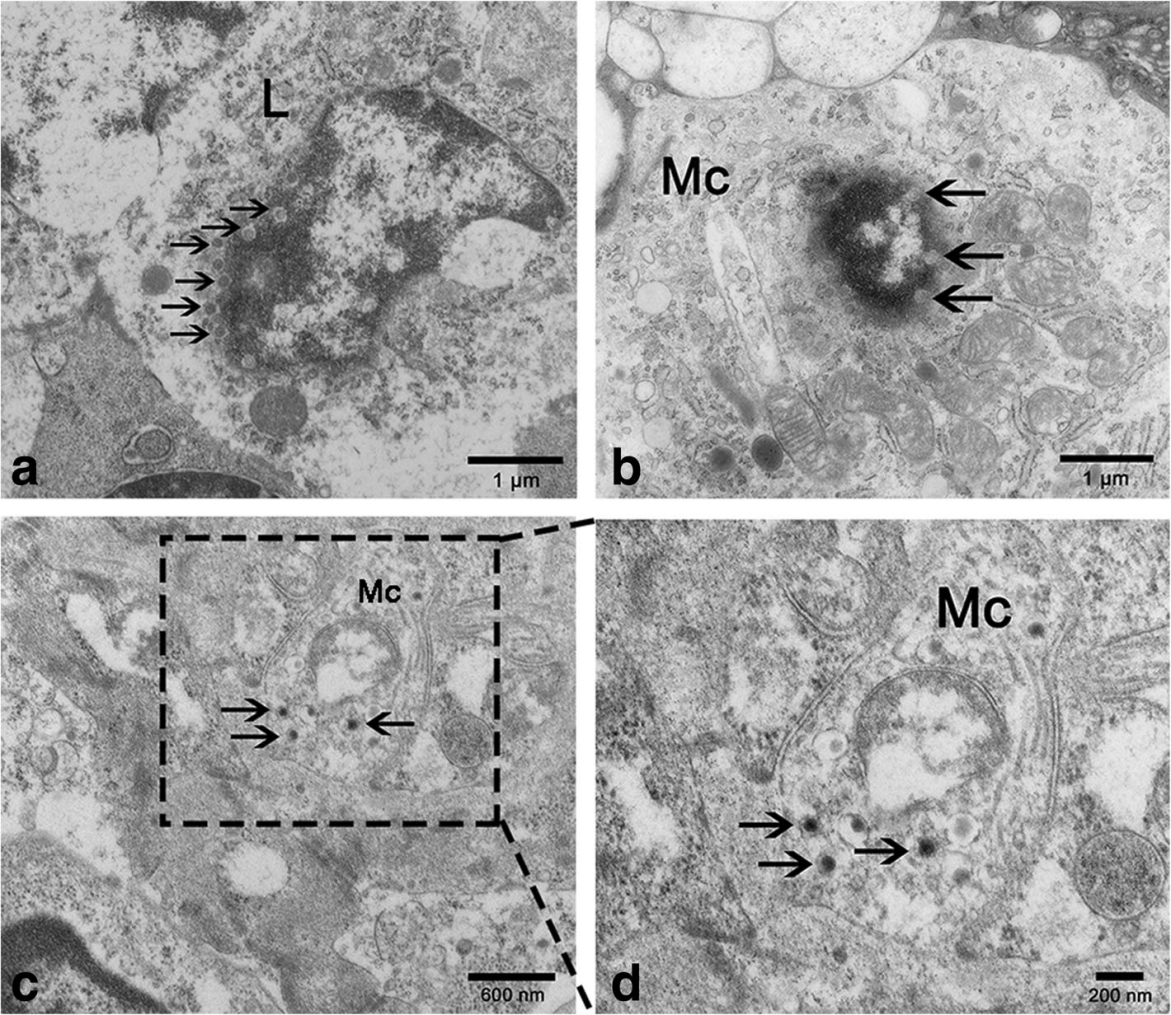
The virus particles in duck spleen under TEM. **a**-**b** At 2hpi and 3hpi, a few virus particles (arrow) without the envelope protein are present in the nucleus and at the surface of lymphocyte nucleus and macrophage nucleus. Bar = 1 μm, 1 μm. **c**-**d** At 9dpi, the intact virus particles are present in the damaged macrophage. Bar = 600 nm, 200 nm. L: lymphocyte; Mc: macrophage

The ultrastructural images revealed that the DTMUV particles were mainly present in lymphocytes and macrophages (Fig. 8). At 2hpi and 3dpi, a few virus particles without E protein were observed on the nucleus surface of lymphocyte and macrophage (Fig. 8a-b). At 9dpi, the intact viral particles were present in the cytoplasm of cells with a damaged nucleus (Fig. 8c-d).

## Discussion

The *Flavivirus* genus, in the *Flaviviridae* family, contains more than 70 viruses [28, 29]. *Flavivirus* usually causes systemic viral injury and chronic disease after infecting humans or mammals due to their spreading through blood and causing viremia. DTMUV, as a novel discovered member of *Flavivirus* genus, has the same pathogenicity with other family members [26]. In this study, the DTMUV was detected in blood at 1 hpi, being present in blood until 18dpi, which indicated that the XZ-2012 could cause viremia for a long time and have a longer course of the disease.

The process of *Flavivirus* infection of hosts usually goes through three phases: initial infection and spread, peripheral viral amplification and neuro invasion [30]. In early infection stage, the virus mainly duplicates locally and causes viremia for the first time. Subsequently, the virus spread into the peripheral organs or tissues of the body and proliferates in large quantities and causes the second-time viremia. Eventually, the virus invades the blood-brain barrier [31, 32]. In this study, DTMUV was detected in spleen tissue from 2 hpi and reached the highest concentration at 1dpi and 3dpi, then reduced and eliminated until 24dpi. Moreover, the *Flavivirus* invades host cells by receptor binding and endocytosis, followed by RNA releases, replicates and assembles new virus particles [33]. According to the process and pattern of other *Flavivirus* infections, the dynamic distribution of virus XZ-2012 in duck spleen could be divided into several stages in our research. After the invasion, the DTMUV caused first-time viremia from 1hpi and accumulated in the spleen through blood circulation at 2hpi. Afterwards, the virus concentration was increasing until 3dpi due to RNA replication and proteins assembly, reaching a replication peak between 1dpi to 3dpi. After 3dpi, the virus concentration was decreased due to the blood spreading into other organs. However, the virus concentration had a slight rise at 12dpi and then recovered, which might suggest the virus returned into the spleen.

The blood-spleen barrier (BSB) as the immune barrier of the spleen is present in the ellipsoid and PELS of white pulp of duck spleen. It relies on the endothelial cells of the sheathed capillary, reticular cells and fibers to block the pathogens in the ellipsoid firstly. Then the ellipsoid-associated macrophages in the PELS could trap antigens in ellipsoids and migrate into PALS through the red pulp finally [27]. The immunofluorescence revealed that the DTMUV appeared in the endothelial cells of the sheathed capillary, the PELS of white pulp and the periphery of the artery at virus invasion and replication phase, which was consistent with the previous research [34]. The ultrastructural results showed that virus particles without the envelope protein were mostly accumulated at the nucleus surface of lymphocytes and macrophages. DTMUV have the tropism of immune cells such as lymphocytes and macrophages. In our recent study, we have found that DTMUV mainly caused vacuolar degeneration of macrophages and lymphocytes in PELS [35]. The structure of the barrier was destroyed. Accordingly, DTMUV might invade the spleen through BSB and replicate in the nucleus of lymphocytes and macrophages after removing the envelope protein. It was considered that lymphocytes, macrophages and endothelial cells could be vulnerable to virus invasion at this phase. At the later phase of infection, the DTMUV particles appeared in PALS around the central artery and the number of viral particles increased significantly at 12dpi compared to that of the control (C). Meanwhile, we also have found that the phagocytosis of macrophages recovered. The macrophages assembled in PELS and subsequently migrated into PALS [35]. The location was consistent with that of BSB. It was speculated that the macrophages recovered its immune function at this moment and played an important role on swallowing virus. The remaining viruses that were not killed by other organs might be returned into the spleen for immune response. However, further experiments are needed to verify how macrophages kill viruses.

Due to various adaptabilities to different host cells for culturing, the value of TCID_50_ was different. There is a variety of infection pathways, including subcutaneous injection, intravenous injection, peritoneal infection and cranial cavity infection. Wu et al. have found that virus titer reached the highest level at 1dpi in spleen after cultured DTMUV PTD2010 strain with duck embryo fibroblast and injected 10^3^TCID_50_ dose [26]. While in this experiment, BHK-21 cells were used for virus culturing and TCID_50_ detection. Using 10^4^TCID_50_ injection dose, the virus XZ-2012 strain content in spleen reached the highest level at 3 dpi. Therefore, there are probably significant differences in the pathological processes due to the different viral doses and strain.

## Conclusion

In summary, this is the first study on the dynamic distribution and content of DTMUV strain XZ-2012 in infected duck spleen from invasion to elimination. It was found that DTMUV invades and replicates in the white pulp of duck spleen via invasion of BSB. It could provide information for the distribution of viruses in spleen from invasion to elimination and the relationship between virus and immune barrier.

## Methods

### DTMUV strain XZ-2012 amplification

DTMUV strain XZ-2012 was isolated and cultured in BHK-21 cells [20]. The virus, freeze-thawed three times, was injected in the allantoic cavity of 10-day specific pathogen free (SPF) duck embryos (200 μl of each). The allantoic fluid was collected after 3–4 days post injection (dpi) and detected by RT-PCR. RNA was extracted with the Trizol method (TransGen Biotech, China) and the quality was assessed using an ND-1000 spectrophotometer (Thermo, USA). RNA samples with an OD260/280 ratio between 1.8 and 2.0 were reverse transcribed into cDNA according to the manufacturer’s instructions (ABM, China). Based on the E gene of XZ-2012 strain in Genbank (Accession No.: KM188953), the primers were designed, forward primer sequence 5′-GAAGCGAGCACCTACCACA-3′, and reverse primer sequence 5′-CGCTGATGACCCTGTCCAT-3′. The expected amplified fragment size was 249 bp. 20 μl reaction volume of PCR: Prime STAR Max Premix (2×) (Takara BioTech, China) 10 μl, forward and reverse primer 0.25 μM, cDNA 1 μg, ddH_2_O 8 μl. The amplification condition: 95 °C 3 min; 95 °C 30 s, 60 °C 30 s, 72 °C 30 s, 35 cycles; 72 °C 10 min. The amplification products were detected by 1% agarose electrophoresis and the positive samples were preserved at − 80 °C for further experiments.

### Virus titration

Viral titers were detected by the TCID_50_ method (50% tissue culture infective dose) using indirect immunofluorescence for detecting virus-induced changes in the cell culture. The BHK-21 cells were cultured in 96-well plates with high glucose DMEM (Dulbecco’s modified eagle medium) including 10% fetal bovine serum (FBS) and 1% penicillin-streptomycin. When the confluence was 80–90%, the cells were infected with 100 μl of serially diluted allantoic virus 10-fold from 10^− 1^ to 10^− 10^ with serum-free DMEM (eight parallel-group of each dilution). At one-hour post infection (hpi), the supernatant was discarded. Then the cells were cultured with maintenance medium including 2% FBS and 1% penicillin-streptomycin for 48 h at 37 °C. After discarding the supernatant, the cells were fixed with 4% paraformaldehyde for 30 min and then incubated with 0.2% Triton X-100 for 20 min to allow permeation. After blocking with 5% bovine serum albumin (BSA) for 1 h at 37 °C, the cells were incubated with the rabbit anti-DTMUV XZ-2012 E protein polyclonal antibody (Homemade polyclonal antibody) for 1 h at 37 °C. After being washed three times with phosphate-buffered saline (PBS), the cells were incubated for 1 h with Alexa Fluor 488 affinipure goat anti-rabbit IgG (H + L) (Fcmacs Biotech Co., Ltd., China). Samples were observed under an inverted fluorescence microscope (Olympus, Japan). The TCID_50_ was calculated using the method of Reed-Muench [36].

### Infection of experimental animals

120 healthy egg-laying shelducks (Nanjing Zhushun Biotechnology Co., Ltd., China), weighting 1.5–2 kg and six months old, negative of any DTMUV and their antibody in vivo using RT-PCR and ELISA (Jiangsu Meimian industrial Co., Ltd., China) were investigated in this experiment. The ducks were divided into one control group and 11 treatment groups (10 per group). The ducks from the treatment groups were injected into leg muscle with the dose of 10^4^ TCID_50_ of the virus and euthanized at different times 1, 2, 6, 12 h and 1, 3, 6, 9, 12, 18, 24 days post-infection (pi). The ducks in the control group were euthanized after injection with the same volume of 0.9% NaCl. The blood samples of each duck were collected and anticoagulated with 1.5% EDTA-Na_2_ and centrifuged to isolate the blood plasma. The spleen samples were fixed in 4% buffered paraformaldehyde and frozen in liquid nitrogen respectively. The virus levels in blood and spleen were detected by RT-PCR and ELISA.

### Quantitative detection of DTMUV in the spleen

#### RT-qPCR detection

RNA of the duck spleens was extracted and reverse transcribed into cDNA. The sequence of amplified target gene was same with the amplified gene sequence of the above RT-PCR experiment (249 bp). Purified PCR products using gel extraction kit (TransGen Biotech, China) were TA cloned into the T-easy vector (FCNCS Tech Co., Ltd., China) and transformed into the DH-5α *Escherichia coli* competent cells (Takara BioTech Co., Ltd., China) according to the manufacturer’s instructions, then cultured overnight onto LB (Luria Bertani) plates with 100 μg/mL ampicillin. After being cultured overnight in liquid LB medium supplemented with 100 μg/mL ampicillin, the ampicillin-resistant transformants were identified by PCR and DNA sequencing. Positive plasmids were isolated according to the instructions of Plasmid Miniprep Kit (TransGen Biotech, China) and quantified using the ND-1000 spectrophotometer. Plasmid copy numbers were calculated using the Avogadro’s constant (6.02 × 10^23^) and prepared according to a tenfold dilution series from 10^1^ copies to 10^9^ copies. The procedure of absolute qPCR was performed according to the instructions of SYBR Green MasterMix-Low ROX Kit (ABM, China) and using Applied Biosystems 7500 Real-Time PCR Systems (ABI, US). The size of the interest sequence was 157 bp and the used primer sequences were as follows:

Forward primer: 5′-AAGCGAGCACCTACCACA-3′,

Reverse primer: 5′-TGCCCCATATCAACTCCAGA-3′.

Each sample was tested in triplicate. The initial virus concentrations were analyzed according to the generated standard curve.

#### Elisa

DTMUV in the blood and spleen homogenate supernatant was quantified by using a commercial double antibody sandwich ELISA kit (Jiangsu Meimian industrial Co., Ltd., China). The ELISA procedure was performed according to the manufacturer’s instructions. The blood plasma and spleen homogenate supernatant were diluted and determined using a standard curve. There were three replicates for each sample. The optical density (OD) values were measured by microplate reader (Bio-Rad, USA).

### DTMUV localization in the spleen

#### Immunofluorescence

The spleen samples, fixed in 4% paraformaldehyde for 24-48 h, were embedded in paraffin, and sectioned to a 3 μm thickness using a Leica microtome (Germany). Briefly, after deparaffinization and washing with PBS, the antigen sections were exposed to citric acid buffer at 121 °C for 10-15 min. Then, they were inactivated with endogenous peroxidase by covering them with 3% hydrogen peroxide for 10 min. After being washed with PBS, the sections were blocked with 5% BSA for 1 h at 37 °C and incubated with the rabbit anti-DTMUV XZ-2012 E protein polyclonal antibody (Homemade polyclonal antibody) for 16 h at 4 °C. After washing with PBS, the sections were incubated for 1 h with Alexa Fluor 488 affinipure goat anti-rabbit IgG (H + L). After washing, the sections were stained with DAPI (4′, 6-diamidino-2-phenylindole) (Boster Biotech, China) for 5 min in the dark. Then the samples were covered and observed with a fluorescence microscope (Olympus DP73, Japan).

#### Transmission electron microscopy (TEM)

The spleen samples were cut into 1 mm^3^ blocks, immersed in 2.5% glutaraldehyde fixative in 0.01 M phosphate-buffered saline (PBS; pH 7.4) at 4 °C overnight, and then submerged in 1% osmium tetroxide in the same buffer for 60 min. The samples were dehydrated in increasing concentrations of ethyl alcohol, infiltrated with a propylene oxide-araldite mixture, and embedded in Araldite. Ultrathin sections (50 nm) were stained with uranyl acetate and lead citrate for 20 min each.

## Abbreviations

BSA: Bovine serum albumin
BSB: Blood-spleen barrier
DMEM: Dulbecco’s modified eagle medium
dpi: day post-infection
DTMUV: *Duck Tembusu virus*
ELISA: Enzyme-linked immunosorbent assay
FBS: Fetal bovine serum
hpi: hour post-infection
LB: Luria Bertani
OD: Optical density
PALS: Periarterial lymphatic sheaths
PBS: Phosphate-buffered saline
PELS: Periellipsoidal lymphatic sheaths
RT-LAMP: Reverse transcription-loop-mediated isothermal amplification
RT-PCR: Reverse transcription-PCR
RT-qPCR: Real-time quantitative PCR
TCID_50_: 50% tissue culture infective dose
TEM: Transmission electron microscopy
